# The Gene Ontology Resource: 20 years and still GOing strong

**DOI:** 10.1093/nar/gky1055

**Published:** 2018-11-05

**Authors:** The Gene Ontology Consortium

## Abstract

The Gene Ontology resource (GO; http://geneontology.org) provides structured, computable knowledge regarding the functions of genes and gene products. Founded in 1998, GO has become widely adopted in the life sciences, and its contents are under continual improvement, both in quantity and in quality. Here, we report the major developments of the GO resource during the past two years. Each monthly release of the GO resource is now packaged and given a unique identifier (DOI), enabling GO-based analyses on a specific release to be reproduced in the future. The molecular function ontology has been refactored to better represent the overall activities of gene products, with a focus on transcription regulator activities. Quality assurance efforts have been ramped up to address potentially out-of-date or inaccurate annotations. New evidence codes for high-throughput experiments now enable users to filter out annotations obtained from these sources. GO-CAM, a new framework for representing gene function that is more expressive than standard GO annotations, has been released, and users can now explore the growing repository of these models. We also provide the ‘GO ribbon’ widget for visualizing GO annotations to a gene; the widget can be easily embedded in any web page.

## INTRODUCTION

**Figure 1.**
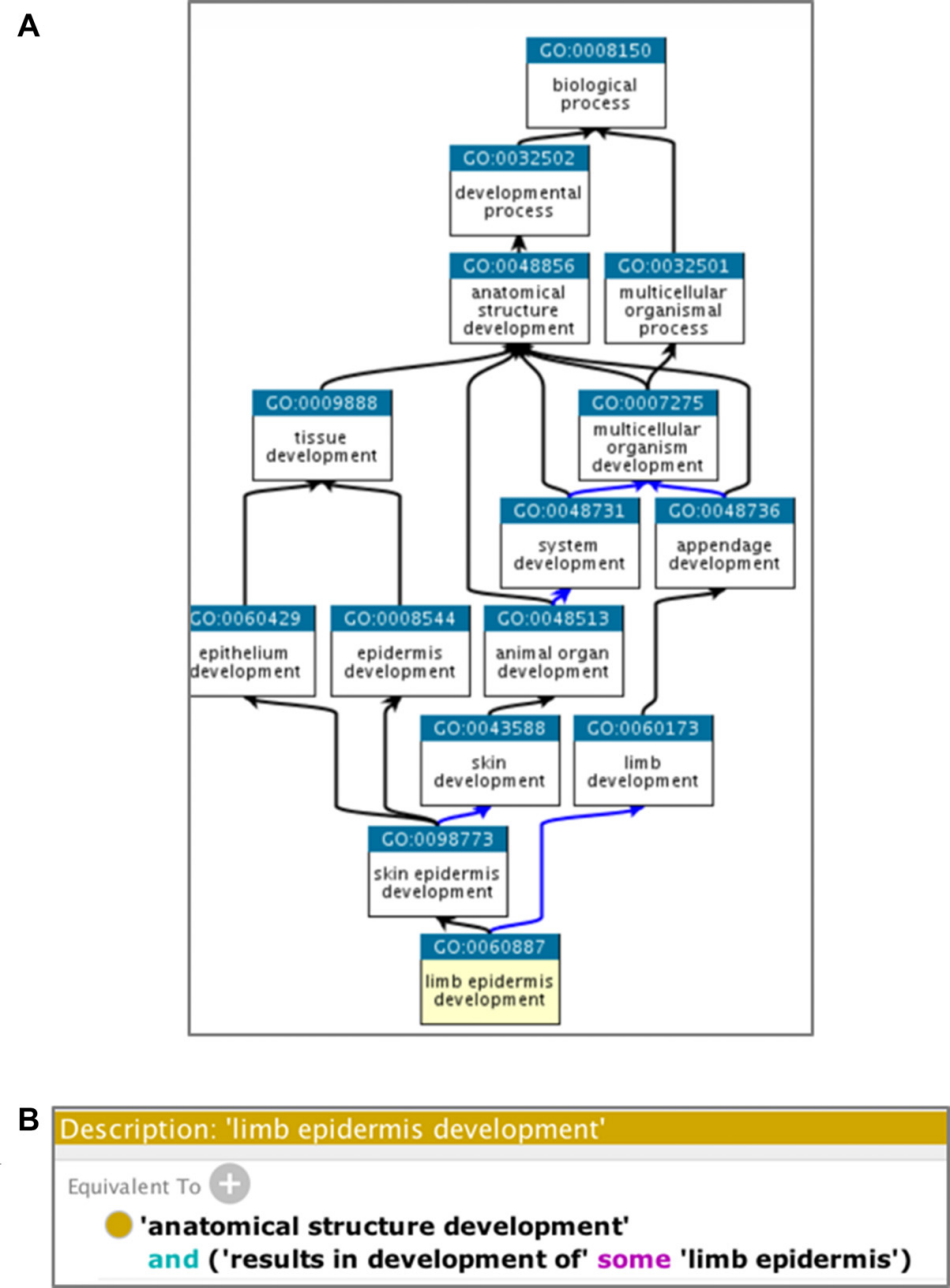
GO structure. (**A**) Graphical representation of relationships between terms: black lines represent *is_a* and blue lines represent *part_of* (representation obtained from https://www.ebi.ac.uk/QuickGO/term/GO:0060887). (**B**) Equivalence axiom for the term ‘GO:0060887: limb epidermis development’, as displayed in Protégé (12).

The Gene Ontology resource (GO; http://geneontology.org) is the most comprehensive and widely used knowledgebase concerning the functions of genes. In GO, all functional knowledge is structured and represented in a form amenable to computational analysis, which is essential to support modern biological research. The GO knowledgebase is structured using a formal ontology, by defining *classes* of gene functions (GO terms) that have specified *relations* to each other (Figure 1A). GO terms are often given logical definitions, or equivalence axioms, that define the term relative to other terms in the GO or other ontologies, so that their relationships can be computationally inferred using logical reasoning (Figure 1B). The GO structure has been meticulously constructed over the course of 20 years by a small team of ontology developers; it is constantly evolving in response to new scientific discoveries and continuously refined to represent the most current state of biological knowledge. The members of the ontology development team are expert biologists and knowledge representation specialists who read the scientific literature and engage biocurators and biological domain experts to collaboratively develop this representation of biological information.

We present here the most important updates since our last contribution to this series (1). There are currently over 45 000 terms in the ontology, linked by almost 134 000 relations. The ontology covers three distinct aspects of gene function: molecular function (the activity of a gene product at the molecular level), cellular component (the location of a gene product's activity relative to biological structures), and biological process (a larger biological program in which a gene's molecular function is utilized).

The GO knowledgebase also includes *GO annotations*, created by linking specific gene products (from organisms across the tree of life) to the terms in the ontology. Each annotation includes the evidence it is based upon, such as a peer-reviewed publication, using evidence codes from the Evidence and Conclusion Ontology (ECO) (2). For example, in its simplest form (what we refer to as a *standard annotation*), an annotation might state that ‘human *MSH2* (a gene, HGNC:7325, also represented by UniProtKB:P43246) is involved in ‘GO:0006298 DNA mismatch repair’ (a GO term), based on a ‘ECO:0000314 direct assay evidence used in manual assertion’ reported in (4)’. Formally, this annotation would be represented in the knowledgebase as a ‘triple’ linking the gene to the GO term using a specific relation: UniProtKB:P43246 *involved_in* GO:0006298. The GO knowledgebase contains over 7 million annotations to genes/gene products from over 3,200 species (http://amigo.geneontology.org/amigo/search/annotation), ∼10% of which (750 000) are supported by experimental data from research papers. Nearly half of these 750 000 experimental annotations refer to genes in a relatively small number of ‘model’ organisms, listed in Table 1. These annotations are made by a consortium of expert biocurators located worldwide, who read scientific papers, ensure the correct gene is identified, and select the most accurate and meaningful GO terms to describe the biology supported by the experimental findings. The accuracy of the GO resource is continually refined by internal checks as well as feedback from the broader GO user community to identify and fix potentially incorrect or out-of-date annotations. The wealth of experimental knowledge manually curated by biocurators is further enriched by inferences from various predictive methods, both manual and automatic, described using classes from ECO as described in (3). In most cases, these annotations are inferred from one or more experimental annotations to a homologous gene product. These may be individually reviewed by a biocurator [denoted by ‘ECO:0000250 sequence similarity evidence used in manual assertion’ (ISS) or ‘ECO:0000318 biological aspect of ancestor evidence used in manual assertion’ (IBA) evidence classes] or not reviewed [denoted by ‘ECO:0000501 evidence used in automatic assertion’ (IEA)].

**Table 1.** Number of experimental annotations in the GO knowledgebase, 5 September 2018 release (doi:10.5281/zenodo.1410625)

	Protein binding, EXP	Molecular function EXP, excluding protein binding	Cellular component EXP	Biological process EXP
**Human**	83589 (+158%)	29999 (+26%)	41341 (+13%)	47230 (+22%)
**Mouse**	12990 (+49%)	14380 (+11%)	28262 (+25%)	67094 (+13%)
**Rat**	4329 (+2%)	10879 (−9%)	15693 (+4%)	27241 (−1%)
**Zebrafish**	509 (+30%)	1756 (+15%)	1087 (+16%)	21635 (+20%)
***Drosophila***	1516 (+33%)	5694 (+15%)	10803 (+3%)	30762 (+1%)
***C. elegans***	2993 (+13%)	2482 (+13%)	5245 (+8%)	14511 (+24%)
***D. discoideum***	690 (+32%)	1081 (+15%)	2738 (+30%)	4149 (+14%)
***S. cerevisiae***	168 (+58%)	8886 (+8%)	17456 (+4%)	20194 (+14%)
***S. pombe***	2076 (+52%)	4636 (+42%)	12184 (+8%)	5651 (+11%)
***A. thaliana***	13074 (+113%)	8344 (+14%)	25486 (+7%)	25223 (+12%)
***E. coli***	3602 (+57%)	6006 (+20%)	4171 (+7%)	5756 (+5%)

Note that for the molecular function annotations, we present annotations to ‘GO:0005515 protein binding’ separately from other GO:0003674 molecular functions, as these GO:0005515 annotations are used differently than other annotations (the class itself is not very informative, but each annotation includes additional information about the specific binding partner). The number of annotations for the main species annotated by the GOC are shown, and the percentage change relative to the 2016 update is indicated in parentheses.

This structure of the GO knowledgebase, the ontology plus annotations, supports queries of the sort that are typically asked in the course of biological research, such as: ‘What are all the functions for the human *ABCA1* gene?’ or ‘What are all the genes involved in the DNA mismatch repair process?’. Because each annotation is associated with evidence (ECO and reference), computer programs can answer even more specific queries, such as ‘What genes have direct experimental evidence of involvement in the DNA mismatch repair process?’, or ‘Which scientific papers provide experimental evidence about the function of the human *ABCA1* gene?’. The ability of the GO knowledgebase to support computational queries is a major reason for its standing as an essential tool in biomedical research. The most obvious example is its use in GO enrichment analysis, also often called pathway analysis (5). For example, a researcher might have identified a set of 1000 genes expressed at a higher level in a cancer sample than in a matched healthy tissue sample, and would like to know if there are any functions (terms from the GO molecular function, cellular component, or biological process aspects) that are unusually common among these 1000 overexpressed genes to understand what may be driving the cancer. To reach this understanding, the functions represented in the set of 1000 genes need to be compared to the functions represented in all 20 000 human protein-coding genes. A computer can use the GO knowledgebase's structure to rapidly retrieve the all the functions that are performed by each of the 20 000 human genes, and create all possible groupings by functional class. Each grouping is tested for statistical enrichment, and the small number of enriched functional classes enables the researcher to identify candidate biological processes within the complex experimental measurement of 20,000 genes.

### GO resource content

The GO knowledgebase consists of the ontology and the annotations made using the ontology. As of the 5 September 2018 release (doi:10.5281/zenodo.1410625), there were ∼45 000 terms in GO: 29 698 biological processes, 11 147 molecular functions and 4201 cellular components, linked by almost 134 000 relationships. The number of annotations (as well as the percentage change since 2016 (1)) are shown in Table 1. It is important to understand that the changes reflect two distinct processes: addition of annotations based on new evidence, and obsoleting of annotations that have been superseded by newer studies. We expect the number of obsoleted annotations to increase, due to our increasing annotation quality assurance efforts, described in more detail below.

#### New framework and repository, for gene function ‘models’

We have developed a more expressive computational framework for representing gene functions, which subsumes our current GO annotation framework, while maintaining compatibility. We refer to the framework as GO-Causal Activity Modeling (GO-CAM, formerly referred to as ‘LEGO’ (1)) and to GO-CAMs as *models* to distinguish them from *standard annotations*. A paper detailing GO-CAM is in preparation, but we summarize some properties here. In GO-CAM, each model is represented as a set of triples (**subject**-*relation*-object, with brackets {} as a set container), e.g. {**ABCA1***enables*cholesterol transporter activity; **cholesterol transporter activity***occurs in*plasma membrane, and **cholesterol transporter activity***part_of* cholesterol homeostasis}. Each triple is supported by one or more pieces of evidence, consisting of a class from ECO and a citable source, usually a scientific publication. GO-CAM specifies the semantics of GO annotations, and how standard GO annotations can be combined into a larger model. Each GO-CAM model is represented using the Web Ontology Language (OWL), which is converted computationally to standard GO annotations (GAF format), ensuring backward compatibility. Users can browse, view, and download the available models in different formats at: http://geneontology.org/go-cam. The number of models is currently small, and most models contain only one gene product (standard annotations extended with additional contextual information, such as cell type). The GO is currently rapidly increasing the curation of GO-CAM models, and the model repository is growing. In particular, models are now available that each represent an entire regulatory or metabolic pathway.

#### Changes to data access: new production pipeline

Starting in March 2018, the releases of the GO resource have been generated by a new data production pipeline using a refreshed software stack. This system allows for easily extensible error checking and improved reporting of quality assurance checks that ensure the quality and integrity of the released ontology and annotations. For users, one of the most important aspects of this pipeline is that the GO resource now produces monthly releases (named by release date) that are available at the GO site and can be referenced and obtained as stable Document Object Identifiers (DOIs) via Zenodo. This feature is critical for ensuring that GO-based analyses can be replicated in a consistent and referenceable manner through the inclusion of these DOIs and/or version of both the ontology and annotation files used (6). Our data production pipeline is currently hosted at the Lawrence Berkeley National Laboratory. We provide GO annotations in multiple formats: as standard GAF (Gene Association Format) and GPAD (Gene Product Association Data) annotation files, in Turtle (OWL serialization format) [http://current.geneontology.org/products/ttl], and a Blazegraph [http://current.geneontology.org/products/blazegraph] journal, replacing the legacy MySQL output.

### Interactions with the GO user community

GO is an open project, and we encourage community contributions to the knowledgebase and software.

**All users**: GO can be contacted using the GO Helpdesk (http://help.geneontology.org) for any questions or feedback about the annotations, the ontology, software, or other GO resources. If users notice an annotation that may not be correct, they should first review the original publication or data source. If the annotation still seems inaccurate, users are encouraged to report this to the GO helpdesk, and GOC members will review the annotation and remove or modify it if justified. **Authors**: Authors can now see if their paper has been used for GO annotation directly in PubMed. Under the ‘LinkOut - more resources’ section of the PubMed abstract page, papers with annotations will have a link labeled ‘Gene Ontology annotations from this paper - Gene Ontology’ (see, e.g. https://www.ncbi.nlm.nih.gov/pubmed/3357510 which links to a web page on the GO site that shows the annotations based on evidence in that paper). If no GO LinkOut is present, that may indicate that the publication has not been used for GO annotation. Authors can contact the helpdesk at the GO website to suggest new annotations or changes to existing annotations. **Resources or consortia**: The GOC collaborates with established data resources and other groups and consortia representing a particular area of biology. Recent examples include cilium biology (7,8), autophagy (9) and cardiac phenotypes (10). We encourage members of other interest groups to contact us to improve the ontology and annotations in their areas of expertise. **Tracking all contributions:** Most aspects of the GO project management are now based in GitHub (https://github.com/geneontology). In addition to tracking ontology change requests (https://github.com/geneontology/go-ontology), we now use GitHub to collect feedback on annotations (https://github.com/geneontology/go-annotation). For users familiar with GitHub, we encourage them to submit any requests directly to GitHub, where they can follow all further discussion and actions. Otherwise, issues and queries can be submitted to our helpdesk (http://help.geneontology.org).

### Increased focus on annotation quality control

The GO resource is now 20 years old. The longevity of the resource adds a challenge to maintain and update the many existing annotations, as many of the findings published during that time have become much more precise, or were reinterpreted or superseded. We have made it a high priority to identify and correct inaccurate and out-of-date legacy annotations to make sure that GO continues to consistently reflect current knowledge. We have taken a number of different approaches to tackle this challenge. First, to ensure consistency and quality, GO biocurators meet regularly for training, establishment of annotation guidelines, and coordinated review of specific areas of biology. More recently, we have made significant efforts to integrate annotation review with ontology improvements, taking advantage of suggested changes to the ontology to clarify term definitions, intended usage, and coordinate annotation practices with curators. In addition, quality assurance is performed centrally, both computationally, to ensure annotations are valid, and manually, to ensure they accurately represent the experimental findings.

We have discovered that one of the most powerful approaches to quality control and consistency is the phylogenetic approach. Originally developed as a means of propagating annotations from experimentally studied genes to evolutionarily related genes in other species, the phylogenetic perspective provides a unified view of all experimental annotations within a evolutionarily-related protein family, allowing curators to more easily find outlier annotations (see e.g. (11)). In parallel, the development of GO-CAM models has been useful in identifying inconsistent annotation practices, and has provided opportunities to develop consortium-wide annotation guidelines. Another observation that emerged is that older annotations from isolated phenotypic observations, taken outside of other contextual data, often do not provide evidence of direct involvement of a gene in a biological process. If inconsistencies are noticed, they are reported to the contributing group for verification and correction as appropriate.

In a pilot quality assurance effort, we have requested the review (by GO Consortium biocurators) of ∼2500 manual annotations (<0.01% of the total corpus) that were judged questionable by one of the strategies above. Approximately 70–80% of the annotations flagged for review were modified to a more appropriate term or removed. We will continue to work on improving the quality of the annotations and reviewing legacy data when appropriate. As a result, we expect that the increase in annotations and new ontology terms may not be as rapid as in the past, at least for the main species annotated by the consortium members, as a greater proportion of our efforts will be dedicated to reviewing and revising older annotations.

### Ontology revision and integration

Since our last update article, we have developed an entirely new process for ontology editing and maintenance that has dramatically increased efficiency and enabled extensive real-time quality checks. Ontology editing is now performed in an OWL-based environment using the ontology editing tool Protégé (12). The ontology is versioned and tracked using a GitHub repository (https://github.com/geneontology/go-ontology). One major advantage of the new ontology management process is that the work can be parallelized among multiple editors, thus increasing efficiency. In addition, real-time quality checks prevent errors that would otherwise require revisiting the same editing task more than once to correct them.

GO continues to integrate and align with external ontology resources in two main ways: import of sub-ontologies used to define GO terms, and inclusion of external cross-references. GO utilizes the structure of external ontologies to aid in reasoning and in the automatic inference of relations between GO terms (13). GO imports subsets of these external ontologies that include information about anatomical structures, cell types, chemicals and taxonomic groupings: Uberon (14), Protein Ontology (15), Plant Ontology (16), ChEBI (17), Relations Ontology (18), NCBI Taxonomy (19), Sequence Ontology (20), Ontology of Biological Attributes (http://www.obofoundry.org/ontology/oba.html), Fungal Anatomy Ontology (http://www.obofoundry.org/ontology/fao.html), Phenotypic Quality Ontology (http://obofoundry.org/ontology/pato.html), and Common Anatomy Reference Ontology (http://www.obofoundry.org/ontology/caro.html). GO also maintains cross-references between terms and multiple widely-used external resources, including Reactome (21), The Annotated Reactions Database (Rhea) (22), Enzyme Commission (EC; http://www.sbcs.qmul.ac.uk/iubmb/enzyme/), IntAct, Complex Portal (23) and MetaCyc (24).

#### Refactoring the molecular function branch of GO

**Figure 2.**
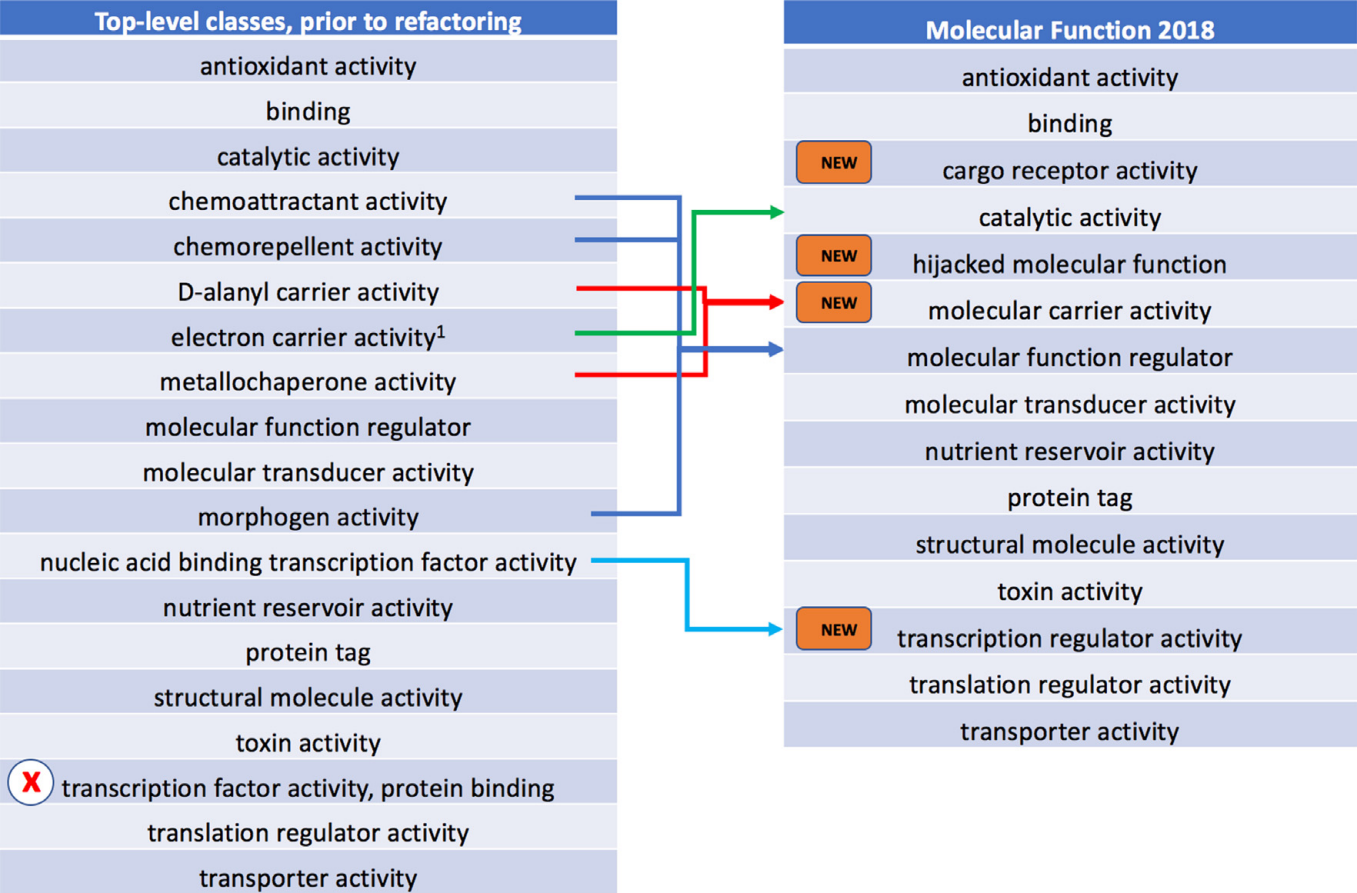
The *molecular function* branch, before and after refactoring. The term marked with an ‘x’ in the left-hand panel has been obsoleted. Terms moved (assigned to a new parent) are indicated by arrows. New terms (right panel) are marked with ‘NEW’. ^1^The class label ‘electron carrier activity’ was changed to ‘electron transfer activity’.

Previously, there was a trend in GO molecular function ontology development to focus on adding terms that describe molecular binding activities of specific gene products. The advantage of such terms is that annotations can often be made unequivocally based on results from a single experiment. However, this approach has led to a complex ontology structure and a proliferation of annotations that individually represent only a partial functional description of a gene product. Annotations to binding terms can obscure annotations to more informative function terms, making annotations more difficult to interpret. For example, one can annotate *CDK1* (UniProtKB: P06493) separately with ‘GO:0030332 cyclin binding’, ‘GO:0005524 ATP binding’, ‘GO:0005515 protein binding’, and ‘GO:0004674 protein serine/threonine kinase activity’. However, these are all aspects of a more precise molecular function that is more informative than the sum of these parts: ‘GO:0004693 cyclin-dependent protein serine/threonine kinase activity’. In the GO molecular function refactoring, we recognized that, while specific binding events are an essential mechanism by which gene products function, an individual binding activity is almost never sufficient in itself to describe molecular function in a larger biological context (25). One of the primary goals of our refactoring was to ensure that the ontology contains the terms necessary to describe these higher-level functions, and that they have a path to the root of the ontology that is not simply under the generic ‘GO:0005488 binding’ term. Accordingly, we reinstated some previously obsoleted terms and added new terms, as well as additional relations. We also addressed the structure of the ontology so that the upper-level terms would be more biologically meaningful and have more uniform specificity. We removed 8 terms from the top level and added four new terms (Figure 2). Most of the terms that were formerly direct children of ‘GO:0003674 molecular function’ were moved under more biologically meaningful terms: for example, ‘GO:0042056 chemoattractant activity’ and ‘GO:0045499 chemorepellent activity’ were moved under ‘GO:0048018 receptor ligand activity’, while ‘GO:0036370 d-alanyl carrier activity’ and ‘GO:0016530 metallochaperone activity’ were moved under the new term ‘GO:0140104 molecular carrier activity’ (representing an activity of ‘directly binding to a specific ion or molecule and delivering it either to an acceptor molecule or to a specific location’). We have also created a new term ‘GO:0104005 hijacked molecular function’ as a parent of terms such as ‘GO:0001618 virus receptor activity’, which is, from the standpoint of the protein being annotated, not a normal function, but nevertheless relevant for some of our users.

**Figure 3.**
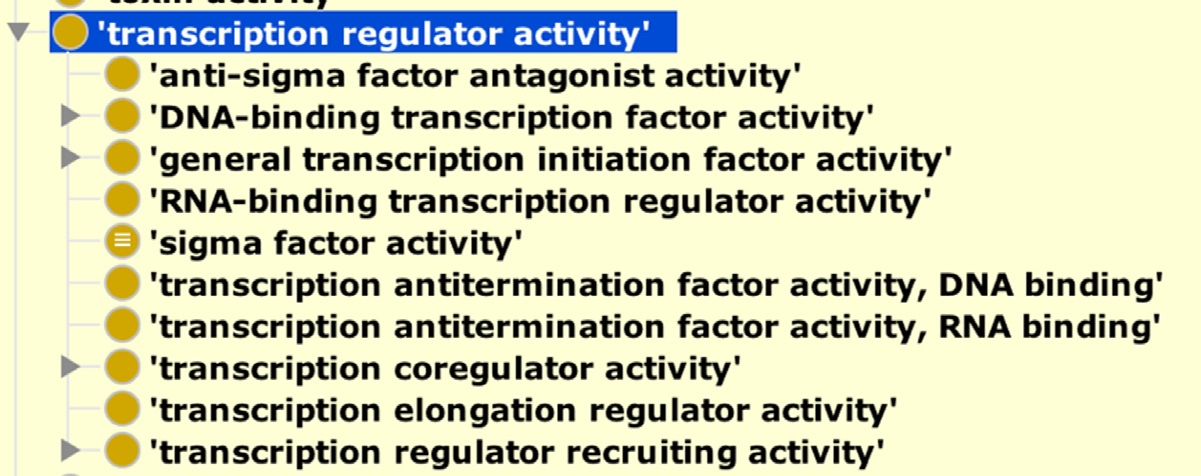
Current structure of the ‘GO:0140110 transcription regulator activity’ branch of the ontology.

Finally, we have made significant changes to the structure representing the molecular functions of transcription factors (Figure 3). This refactoring was carried out in collaboration with experts in transcription factors and gene regulation from the Gene Regulation Consortium (GRECO; http://thegreco.org). In keeping with our design principle of having terms that describe higher-level functions, we have created a new parent term to group all functions that directly regulate transcription, ‘GO:0140110 transcription regulator activity’. The formerly top-level term ‘GO:0000988 transcription factor activity, protein binding’ has been obsoleted because this activity was partly covered by other terms in the ontology and its usage was inconsistent. Accordingly, its children have either been obsoleted or subsumed under different terms (merged or moved). The new top level term ‘GO:0140110 transcription regulator activity’ has three main children - ‘GO:0003700 DNA-binding transcription factor activity’ (formerly labeled ‘transcription factor activity, sequence-specific DNA binding), ‘GO:0140223 general transcription initiation factor activity’ and ‘GO:0003712 transcription coregulator activity’.

The transcription factor areas of GO had previously been refactored between 2010 and 2012 (26,27) with the aim of more finely capturing all combinations of different types of protein and DNA binding activities (e.g. binding to different types of regulatory regions such as promoters and enhancers) and transcription regulation processes (positive and negative regulation). However, this structure, while very precise, has proved very difficult to use by biocurators, resulting in inconsistent annotations. Additionally, end-users have had difficulty with common queries, such as comprehensively identifying the set of all transcription factors in a given species. We expect that even more improvements to the ontology structure, as well as more consistent annotations to transcription regulator terms, will be available in 2019.

#### Defining the boundaries of biological processes: MAP kinase signaling and extracellular matrix as examples

We have used our integrated annotation review and ontology development methodology to refine two areas of the ontology, the MAP kinase signaling pathway and the representation of the extracellular matrix. Refinements to the MAPK cascade included defining the molecular functions that are parts of the process: ‘GO:0004707 MAP kinase activity’, ‘GO:0004708 MAP kinase kinase activity, ‘GO:0004709 MAP kinase kinase kinase activity’ and ‘GO:0008349 MAP kinase kinase kinase kinase activity’. All other upstream and downstream molecular functions/biological processes will be modeled in GO-CAM with causal relationships between them and the MAPK cascade. We also enumerated the types of cascades based on current literature and on discussions among expert model-organism curators, trying to keep the distinctions useful across multiple taxa. There are four direct subtypes of ‘GO:0000165 MAPK cascade’: ‘GO:0070371 ERK1 and ERK2 cascade’, ‘GO:0070375 ERK5 cascade’, ‘GO:0071507 pheromone response MAPK cascade’ and ‘GO:0051403 stress-activated MAPK cascade’. Some other MAPK processes such as ‘GO:1903616 MAPK cascade involved in axon regeneration’ will eventually be obsoleted, as these combine two or more other GO terms and can be composed in GO-CAM. For the refinement of the extracellular matrix area of the ontology, we worked with external experts to add terms that were useful grouping terms such as ‘GO:0062023 collagen-containing extracellular matrix’. We also obsoleted or merged terms that were poorly annotated and thought to represent an outdated view.

#### GO subsets (slims)

A GO subset (or slim) is a set of GO terms selected to provide an overview of the functions, locations or roles of a set of genes. The subset can be developed for high coverage of specific species, or to represent only certain areas of the ontology, and in most cases, contain only high-level GO terms to provide a broad biological overview. Another use of subsets is to blacklist certain terms for annotation: GO has two such subsets, one to flag terms that should not be used for manual annotation, and one for terms that should not be used at all. GO maintains two additional subsets, the Generic GO slim and the Alliance of Genome Resources (https://www.alliancegenome.org/) slim. GO also hosts subsets useful to groups using GO; we currently have 11 such subsets (Table 2; http://www.geneontology.org/page/go-subset-guide). Each subset provides a global overview of gene functions. Each subset now has a designated contact person to resolve any issue resulting from ontology changes (see Ontology revision and integration).

**Table 2.** Subsets maintained in GO

GO subsets	
Generic GO subset	
GO slim AGR	
GO do not annotate list	
GO do not manually annotate list	
**Subsets from external groups**	
**Subset**	**Group**
Aspergillus subset	Aspergillus Genome Data
Candida albicans	Candida Genome Database
Chembl Drug Target subset	ChEMBL
FlyBase Ribbon slim	FlyBase
Metagenomics subset	EBI Metagenomics group
Mouse GO slim	MGI
Plant subset	The Arabidopsis Information Resource
Protein Information Resource subset	PIR
Schizosaccharomyces pombe subset	PomBase
Synapse GO slim	SynGO
Yeast subset	Saccharomyces Genome Database

### Other developments

#### The GO ribbon: a configurable tool for visualizing GO annotations

Many genes have large numbers of annotations, making it difficult to get a quick overview of a gene function, or the functions of gene sets. We have developed the GO ribbon specifically to help users visualize and explore the functions of a gene. The GO ribbon visualization metaphor borrows from a viewer originally developed by the Mouse Genome Database team (28), but in contrast, the GO ribbon was developed as a lightweight, reusable widget that can be embedded in any website, and retrieves data directly from the GO resource via API.

**Figure 4.**
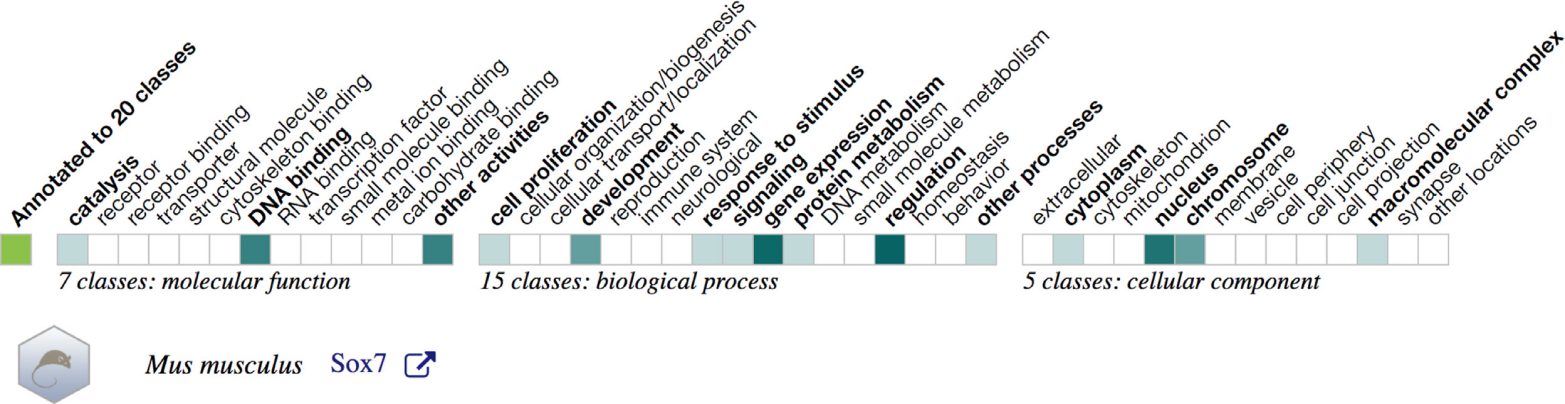
GO ribbon representation. Darker boxes indicate terms with the most annotations; white boxes represent terms that are not annotated for this protein (*Mus musculus* Sox7, MGI:98369). Screenshot obtained from https://www.alliancegenome.org/gene/MGI:98369.

To generate a GO ribbon, all the functions (GO terms) associated with a gene of interest are mapped onto a specified GO subset using the ontology structure. The end result is a simple graphical representation of a gene's functions (Figure 4). The ribbon is interactive, allowing users to drill down to more specific functions by selecting a high-level category such as ‘GO:0030154 cell differentiation’, ‘GO:0050877 nervous system process’, or ‘GO:0003700 DNA-binding transcription factor activity’, and to filter the functions based on the evidence codes provided in the GO annotations. This overview of gene functions is particularly useful when comparing the functions of different genes in the same species, or the functions of orthologous genes across different species.

The GO ribbon is a React component available on GitHub (https://github.com/geneontology/ribbon) and as a NPM package https://www.npmjs.com/package/@geneontology/ribbon). The GO ribbon widget is currently used by the Alliance of Genome Resources.

#### GO annotations from high-throughput experiments

Data from high-throughput experiments are generally collected in a hypothesis-free manner, and consequently do not generally provide as strong evidence of gene function as small-scale molecular biology experiments that currently support most of the experimental GO annotations. In addition, high-throughput experiments can be subject to relatively high false positive rates. Users may therefore want to filter out these experimental annotations in some applications of the GO. To make this possible, starting in 2018, in collaboration with the Evidence and Conclusions Ontology (29) (2), the GO has added several new evidence codes to describe high-throughput experiments: ‘ECO:0006056 high throughput evidence used in manual assertion’ (HTP), and the subclasses: ‘ECO:0007005 high throughput direct assay evidence used in manual assertion’ (HDA), ‘ECO:0007001 high throughput mutant phenotype evidence used in manual assertion’ (HMP), ‘ECO:0007003 high throughput genetic interaction evidence used in manual assertion’ (HGI) and ‘ECO:0007007 high throughput expression pattern evidence used in manual assertion’ (HEP). To accompany the new evidence codes, we have provided annotation guidelines to help identify and curate high-throughput datasets that meet the GO Consortium annotation criteria. Consortium members have reviewed papers with more than 40 annotations using a single evidence code, and updated the evidence codes, or removed the annotations if appropriate. There are currently over 31 000 annotations that have HTP evidence codes from 140 research articles, representing <5% of experimental GO annotations. The identification of annotations derived from high-throughput experiments allows users to choose to exclude these from their analyses, if they are concerned that these annotations may lead to an increased bias in data analysis. This is likely to be particularly important, as is often the case, when GO is used to interpret types of data similar to those on which the annotations are based.

## CONCLUSIONS

The GO resource has been under continuous development for 20 years, with no signs of slowing down. Both the ontology and annotations continue to be updated steadily, in response to new experimental findings concerning gene function, and accumulating knowledge of how genes function together in larger systems. The GO Consortium is increasing efforts to review annotations, especially those that are older and may have been superseded by newer findings. GO has always been an open, community project, and we hope that users of GO will contact us with suggestions for how we can improve the resource. GO releases are now monthly, with persistent DOI’s, and we recommend that all published GO-based analyses cite this DOI, to enable reproducibility. GO-CAM, our new framework for defining and representing gene functions with more accuracy, consistency and precision, is being used to create a growing set of curated biological models, and we encourage the analysis tool developer community to explore the new format and potential new applications of these models.

## Appendix of Authors

**Berkeley Bioinformatics Open-Source Projects (BBOP), Environmental Genomics and Systems Biology Division, Lawrence Berkeley National Laboratory** (Berkeley, CA, USA): S. Carbon*, E. Douglass, N. Dunn, B. Good, N.L. Harris, S.E. Lewis, C.J. Mungall; **dictyBase, Northwestern University** (Chicago, IL, USA): S. Basu, R.L. Chisholm, R.J. Dodson, E. Hartline, P. Fey; **Division of Bioinformatics, Department of Preventive Medicine, University of Southern California** (Los Angeles, CA, USA): P.D. Thomas*, L.P Albou*, D. Ebert, M.J. Kesling, H. Mi, A. Muruganujan, X. Huang, S. Poudel, T. Mushayahama; **EcoliWiki, Departments of Biology and Biochemistry and Biophysics, Texas A&M University** (College Station, TX, USA): J.C. Hu, S.A. LaBonte, D.A. Siegele; **FlyBase, Department of Physiology, Development and Neuroscience, University of Cambridge** (Cambridge, UK): G. Antonazzo, H. Attrill, N.H. Brown, S. Fexova, P. Garapati, T.E.M. Jones, S.J. Marygold, G.H. Millburn, A.J. Rey, V. Trovisco; **FlyBase, The Biological Laboratories, Harvard University** (Cambridge, USA): G. dos Santos, D.B. Emmert, K. Falls, P. Zhou; **FlyBase, Department of Biology, Indiana University**, (Bloomington, USA): J.L. Goodman, V.B. Strelets, J. Thurmond; **GO-EMBL-EBI** (Hinxton, UK): M. Courtot, D. Osumi-Sutherland, H. Parkinson, P. Roncaglia; **Gene Regulation Consortium (GRECO), Norwegian University of Science and Technology** (Trondheim, Norway): M.L. Acencio, M. Kuiper, A. Lægreid; **Gene Regulation Consortium (GRECO), Radboud University** (Nijmegen, The Netherlands): C. Logie; **Institute of Cardiovascular Science, University College London** (London, UK): R.C. Lovering, R.P. Huntley, P. Denny, N.H. Campbell, B. Kramarz, V. Acquaah, S.H. Ahmad, H. Chen, J.H. Rawson; **Institute for Genome Sciences, University of Maryland School of Medicine** (Baltimore, MD, USA): M. C. Chibucos, M. Giglio, S. Nadendla, R. Tauber; **IntAct/Complex Portal, EMBL-EBI** (Hinxton, UK): M.J. Duesbury, N Del-Toro, B.H.M. Meldal, L. Perfetto, P. Porras, S. Orchard, A. Shrivastava, Z. Xie; **InterPro, EMBL-EBI** (Hinxton, UK): H.Y. Chang, R.D. Finn, A.L. Mitchell, N.D. Rawlings, L. Richardson, A. Sangrador-Vegas; **Mouse Genome Informatics, The Jackson Laboratory** (Bar Harbor, ME, USA): J.A. Blake, K.R. Christie, M.E. Dolan, H.J. Drabkin, D.P. Hill*, L. Ni, D. Sitnikov; **PomBase, University of Cambridge** (Cambridge, UK): M.A. Harris, S.G. Oliver, K. Rutherford, V. Wood; **PomBase, The Francis Crick Institute** (London, UK):J. Hayles; **PomBase, University College London** (London UK): J. Bahler, A. Lock; **RGD, Medical College of Wisconsin** (Milwaukee, WI, USA): E.R. Bolton, J. De Pons, M. Dwinell, G.T. Hayman, S.J.F. Laulederkind, M. Shimoyama, M. Tutaj, S.-J. Wang; **Reactome, Department of Biochemistry & Molecular Pharmacology, NYU School of Medicine** (New York, NY, USA): P. D’Eustachio, L. Matthews; **Renaissance Computing Institute, University of North Carolina** (Chapel Hill, NC, USA): J.P. Balhoff; **SGD, Department of Genetics, Stanford University** (Stanford, CA, USA): S.A. Aleksander, G. Binkley, B.L. Dunn, J.M. Cherry, S.R. Engel, F. Gondwe, K. Karra, K.A. MacPherson, S.R. Miyasato, R.S. Nash, P.C. Ng, T.K. Sheppard, A. Shrivatsav VP, M. Simison, M.S. Skrzypek, S. Weng, E.D. Wong; **SIB Swiss Institute of Bioinformatics** (Geneva, Switzerland): M. Feuermann, P. Gaudet*; **TAIR, Phoenix Bioinformatics** (Fremont, CA, USA): E. Bakker, T.Z. Berardini, L. Reiser, S. Subramaniam, E. Huala; **UniProt: EMBL-EBI** (Hinxton, UK), **SIB Swiss Institute of Bioinformatics (SIB)** (Geneva, Switzerland), and **Protein Information Resource (PIR)** (Washington, DC, USA and Newark, DE, USA): C. Arighi, A. Auchincloss, K. Axelsen, G., Argoud-Puy, A. Bateman, B. Bely, M.-C. Blatter, E. Boutet, L. Breuza, A. Bridge, R. Britto, H. Bye-A-Jee, C. Casals-Casas, E. Coudert, A. Estreicher, L. Famiglietti, P. Garmiri, G. Georghiou, A. Gos, N. Gruaz-Gumowski, E. Hatton-Ellis, U. Hinz, C. Hulo, A. Ignatchenko F. Jungo, G. Keller, K. Laiho, P. Lemercier, D. Lieberherr, Y. Lussi, A. MacDougall, M. Magrane, M. J. Martin, P. Masson, D.A. Natale, N. Hyka-Nouspikel, I. Pedruzzi, K. Pichler, S. Poux, C. Rivoire, M. Rodríguez-López, T. Sawford, E. Speretta, A. Shypitsyna, A. Stutz, S. Sundaram, M. Tognolli, N. Tyagi, K. Warner, R. Zaru, C. Wu; **University at Buffalo, Department of Biomedical Informatics** (Buffalo, NY, USA): AD. Diehl; **WormBase California Institute of Technology** (Pasadena, CA, USA), **Wellcome Trust Sanger Institute** (Hinxton, UK), **EBI** (Hinxton, UK), and **Ontario Institute for Cancer Research** (Toronto, Canada): J. Chan, J. Cho, S. Gao, C. Grove, M.C. Harrison, K. Howe, R. Lee, J. Mendel, H.-M. Muller, D. Raciti, K. Van Auken*, M. Berriman, L. Stein, P. W. Sternberg; **ZFIN, University of Oregon** (Eugene, OR, USA): D. Howe, S. Toro, M. Westerfield.

*Authors who contributed significantly to writing this manuscript.
